# Medical Items and News of Interest to the Physician

**Published:** 1877-01

**Authors:** 


### Medical Items and News,

### For the Use of Physicians.

CULLED FROM THE STANDARD MEDICAL JOURNALS OF THE WORLD.

NOTE.—These formulæ are intended only for the regular practitioner of medicine, and should be employed only by him, or under his immediate supervision.

**Antidote to Strychnia.**

The East Indian physicians recommend nicotia as the surest antidote, which is given in exceedingly small quantities in sherry several times a day. In default of nicotia, decoction of tobacco leaves (half an ounce to a pint) is given.—*Amer. Jour. of Pharm.*

**Toothache.**

Dr. Buckworth (*Practitioner*) says that having failed in relieving a case of toothache by all the usual remedies, he succeeded by the use of carbonate of soda. Half a drachm was dissolved in an ounce of water. The patient was directed to hold a spoonful in his mouth, and to his astonishment the pain ceased immediately and complete relief was secured.

**Hemorrhage.**

It has been asserted that bromide of potassium induces contraction of the blood-vessels, and Dr. Geneuil (*L' Union Medicale*) has used a local application in the treatment of epistaxis, uterine hemorrhage, and coryza. It should also be given internally. In coryza two injections of a saturated solution into the nose, give relief in an hour, and a permanent cure is effected in about six hours.

**Obesity.**

The *Paris Medicale* discusses the treatment of obesity by the administration of sea-water combined with a residence at the seaside. Sea-water taken internally, acts like diuretic and purgative salts, a remarkable fact being that the diuretic effect increases when the purgative diminishes. The water should be obtained, when possible, from some depth, and far from shore. It is then to be left to settle for six or twelve hours, and filtered. It is to be taken three times a day, in doses of a small glass full, or in half that quantity at a time, with fresh water or milk. It is stated as a fact that sea-water thus used facilitates the oxygenation of the blood, and that it hastens the elimination of effete materials. In combination with this treatment, sea-water baths are to be taken, free exercise is to be practiced, and fattening foods avoided.

**Neuralgia.**

Dr. J. S. Jewell (*Chic. Jour. Ner. and Ment. Dis.*) strongly recommends for the relief of neuralgic pain the use of a combination of morphia and atropia. The proportion is one grain of the latter to sixteen to twenty-four of the former, to an ounce of distilled water. This is given hypodermically in doses suitable to relieve pain. He says that he knows of no combination of narcotics so valuable in cases of neuralgia, and at the same time attended by so few ill consequences.

**Asthma.**

A writer in the *Peninsular Jour. of Med.* says an analysis of "Langell's Asthma Remedy" shows that it is a mixture of coarsely powdered belladonna leaves with one part of nitrate of potassium dried together. The packages contained about two ounces and were priced at $1.25 each. On igniting a portion on a plate, combustion takes place slowly and the fumes are inhaled. It is said to give prompt relief, is much asked for, and can be sold in quantities for about half a dollar a pound.

**Uterine Catarrh.**

Dr. Gereleitschenko has tried the effect of applying ergot to the canal of the cervix uteri in twenty-nine cases of chronic metritis attended with catarrh. In twenty-two of these the discharge ceased, the swollen state of the mucous membrane disappearing, and the uterus itself becoming smaller and firmer. The subjective symptoms also ceased, and regular menstruation ensued. In the other seven cases the improvement which ensued was soon followed by relapse, the uterus in these cases having undergone more advanced alterations.—*St. Petersburg Med. Woch.*

**Coca.**

Since the writing of Sir R. Christison on his experience in the use of coca leaves as a means of preventing fatigue, several have experimented upon themselves. Among the latest who have published the results are two Irishmen :—Walter Bernard and J. R. Leebody of Londonderry. Starting one night at midnight, after a day of unusual fatigue, they walked twenty-four miles, part of the way over a stony road, and ascended a steep hill over thirteen hundred feet high. Both men, in the course of the walk, chewed each two doses of forty grains of the leaves; and were decided in their opinions that they suffered less from fatigue or the effects of the severe exercise than they would have done had the chewing of the coca not been resorted to.—*Brit. Med. Jour.*

**Neuralgia.**

The *Bordeaux Medical* gives the following formula for obstinate neuralgia, especially ileo-lumbar neuralgia:—

℞

Valerianate of ammonia,
Quinine, āā gr. xxx.

Make into twenty pills and take from two to ten of them each day, increasing one pill per diem. After taking these pills for ten days, suspend their use for five days.

---

**Indication of Death.**

Is the patient really dead or not? is at times a very anxious question. A medical practitioner of Cremona proposes a simple method by which the question may be answered with certainty. It is to inject a drop of ammonia beneath the skin, when, if death be present, no effect, or next to none, is produced; but if there be life, then a red spot appears at the place of the injection. A test so easily applied as this should remove all apprehensions of being buried alive.

---

**Dropsies.**

Dr. J. B. Leary reports, in the *Proceedings of the Med. Soc. of the Co. of Kings*, four cases in which the use of fluid extract of eucalyptus glob., in doses of eight or ten minims as a diuretic, resulted most favorably. While patients have been taking it they sometimes complained of a very severe congestive headache, accompanied with tinnitus aurium; that their appetites were much better, though no tonics were prescribed, and in some cases the fæcal movements were more fluid than usual.

---

**Pigmentary Stains after Venereal Eruptions.**

M. Langlebert wishes to make known a simple means of effecting the removal of the pigmentary spots syphilitic eruptions, and especially ecthyma, leave behind. Of a deep yellow, sometimes almost black, they are very lasting, those on the legs sometimes persisting for years, to the great annoyance of the patients. The various applications hitherto employed being of little avail, M. Langlebert having remarked that blisters which had been kept open only for a few days leave, especially in individuals of a dark complexion, white traces that are well-nigh indelible, applied in an old case of syphilitic ecthyma small blisters over the syphilitic patches, and maintained suppuration for a week by means of blistering ointment. The success was complete, and has induced him to recommend the adoption of the practice.—*Gaz. des Hop.*

**Enlargement of the Spleen.**

Dr. Guild, Jr., of Tuscaloosa, Alabama, writes to the *Med. and Surg. Reporter* that the muriate ammonia, as recommended by Eberle, is the great desideratum. As a resolvent, it has in my hands proved entirely efficacious. It will give speedy relief only when given in purgative doses, thirty or forty grains. I make a saturated solution and give a tablespoonful every two hours. Other very important adjuvants should not be overlooked, such as the iodine ointment, often applied over the left hypochondrium, with an occasional aloetic purge.

---

**New Febrifuge.**

Dr. Brunnetti, of Constantinople, recommends the following mixture as being not only much cheaper but more efficacious than quinine in malarious fevers:—Chloride of sodium, 12 grammes (185 grains); carbonate of iron, 1 gramme (15½ grains); mix and divide into three powders. These three doses are to be taken in one day, one powder being taken in the morning, another at mid-day, and the third at night. In order to prevent a relapse, one powder should be given every morning for eight days. He finds it also superior to other anti-pyretics in tuberculosis, in smaller doses than the above, but continued for a longer time.—*Chemist & Druggist.*

---

**Chronic Intermittents.**

In the *Cincinnati Lancet and Observer*, Dr. L. M. Jones reports that having failed, in the case of a girl aged ten years, in treating a case of chronic intermittent with quinine, camphor, chinodine, pills, arsenic, eucalyptus, iodine, phosphoric acid, etc., he adopted the suggestion of Dr. Jackson in the United States Dispensatory, to use spider-web.

A bunch of spider web collected from a dark cellar (as it is the web of a species of spider which inhabits dark places that possesses medical properties), about the size of a large hulled walnut, was put into four ounces of whiskey and allowed to macerate forty-eight hours, when it was filtered. The patient took a teaspoontul four hours before the expected chill, and at hourly intervals until she had taken four doses, and then took a like quantity before each meal and at bed time until all was taken. Although the anticipated chill came, it was very light, and was the last one experienced up to the time of the report, a period of four months. Neither the patient nor her friends knew the nature of the remedy used, so imagination had no effect on the cure. The remedy has succeeded equally well in subsequent cases.

**Linim. Chloroformi.**

℞

Chloroformi ʒ 2.

Tr. Opii ʒ 2.

Linim. Sapon. fl ℥. 1½ M.

**Buck's Burn Mist.**

℞

Pulv. G. Tragacanthe ʒ 2.

" G. Acaciæ ʒ 4.

Syr. fusci (Molasses) fl ℥ 16.

Aquæ bullient, pint 1. M.

**Lotion for Urticaria.**

Hardy recommends the following lotion to relieve the irritation of urticaria: Chloroform, 10 parts; oil of sweet almonds, 30 parts; to be used several times daily.

**Aortic Aneurism.**

Dr. John Duncan showed recently to the Medico-Chirurgical Society of Edinburgh a case of aortic aneurism consolidated and apparently cured by rest and the use of iodide of potassium.—*Medical Examiner.*

**Salicylate of Ammonium.**

J. F. Martenson (*Centralbl. f. Med.*, No. 19, 1876, p. 352) suggests the use of this salt which is easily made by saturating the aqueous solution of the acid with ammonia or its carbonate. It is easily soluble in water and alcohol, has a sweetish taste, and is tolerably stable. One hundred parts contain about eighty-nine parts of salicylic acid.

**Concentrated Solution of Salicylic Acid.**

℞

Acid salicylic pur., ʒ ij.

Sodii biborat, ʒ j.

Glycerinæ, q. s.

Mix the acid and borax with f ʒ iv. of glycerine, heat gently until dissolved, and then add enough glycerine to make the whole measure one fluid ounce. Tho solution contains 25 per cent. of salicylic acid, and can be diluted with either glycerine, alcohol or water, to any degree required.

**Relief of Cancer.**

A correspondent of the *British Medical Journal*, writing after a visit to the Samaritan Hospital, says: "We saw also, with Dr. W. Williams, a woman aged 50, whose cervix uteri had been amputated for epithelial cancer by Mr. Baker-Brown, eight years before. The actual cautery had been applied later by Dr. Routh, and later still, Dr. W. Williams had injected bromine at three sittings, after which the whole of the affected part came away, and complete healing took place. The parts are now quite sound. There was apparently only an inch of the uterus left. The solution used is one part of bromine to three of rectified spirits. This develops heat, and should be prepared some time before being used. From five to ten minims are injected into the tissues by means of a long syringe with a platinum nozzle and an india-rubber piston. It is desirable to remember that it may destroy the sense of smell in the operator, and that this may be prevented by placing alkalinized cotton-wool in the nostrils."

**Anti-Salivation Powder.—(Panas.)**

℞

Pulv. cinchon cort., 15 grammes.
" g. catechu, 15 grammes.
" acidi tanici, 2 grammes.
Alum. et potass. sulphat, 1 gramme.
Ess. menth. vel. anisi, q. s. M.

The gums are to be well rubbed with this powder several times a day, to prevent or diminish swelling of the gums, in patients using mercury—especially those making inunctions with the Napolitane Ointment.—*Annuaire de Therap.*

**Strumous and Syphilitic Ulcers.**

M. Guillaumet, who has experimented extensively upon the local employment of carbon bisulphide, says, in reply to objections that have been made to its supposed poisonous character, and on account of its smell, that as regards the first, it may be freely inspired without harm, and for the second, that it may be deodorized by peppermint essence and tincture of iodine; the latter augmenting its beneficial effects. He mentions cases in which it has been of value when used in lupus of the face, and in strumous and syphilitic ulcerations of the female generative organs.—*Jour. de Therap.*

**An Emmenagogue Pill.**

The formula for which is given in the *Union Medicale*, may be made as follows:

℞

Aloes, gr. xv.
Rue,
Saffron,
Savin, āā gr. vijss. M.

Divide into 10 pills, one of which is to be taken morning and evening, commencing two or three days before the supposed menstrual epoch. It is desirable to employ, in connection with the pills, warm hip-baths, dry cups to the lumbar regions, leeches to the upper and inner portion of the thighs, and walking exercise; also give in the menstrual intervals, iron and quinia.

**Enlarged Spleen.**

Dr. J. J. Jones, of Little Rock, Ark., reports, in the *Med. and Surg. Reporter*, the case of a man, aged 28, who suffered from malarial fever and an enlarged spleen—so large, indeed, that a physician who saw him in consultation advised Dr. Jones to "detach the man from the spleen, as the best mode of curing it." Fluid extract of ergot was ejected under the skin to the extent of half a dozen doses of eight drops on successive days, with the effect of reducing the spleen to respectable size and curing the patient.

**Sedative Solution in Whooping-Cough.**

℞

Musk, gr, iij.
Potassic bromide, ʒ ss. to ℈ ij.
Cherry laurel water, ʒ iss.
Syrup of Ether, ℥ ss.
Syrup of Belladonna,
Syrup of Codeine, āā ℥ i.
Syrup of Orange flower, ℥ iss. M.

To a child eight or ten years of age, give a teaspoonful between meals, morning, evening and night. During the day it is not to be used, lest the narcotics recommended disturb digestion. The musk, if insupportable, may be omitted.—Gueneau de Mussey, in *Jour. de Med. et de Chir. Pratiques.*

**Pruritus.**

Dr. Boeck, of Christiania, reports the results of the smoke of Juniper needles, in several cutaneous affections, in which itching forms a most distressing symptom, especially urticaria, pruritus and prurigo. The patient is inclosed as for an ordinary mineral vapor bath, and beneath him, with proper precautions against the blaze which may ensue, is placed a pan of live coals, upon which the juniper leaves have been thrown. If not freshly picked, the neeedles should be damped with water. The patient is to remain exposed to the vapor for twenty or thirty minutes, generally on every second day. In purigo, the remedy is immediately effective, and many cases have, after treatment in hospital by this means, been discharged cured. The most marked effects were obtained in bad cases of chronic urticaria and pruritus.

**Preservation of Syrups.**

Mr. Lajoux, a Paris pharmacien, has been making some experiments with the object of ascertaining the minimum quantity of salicylic acid by which the fermentation of syrups can be prevented during the summer. The syrups experimented upon were red currant, cherry, mulberry, capillaire, gentian and compound ipecacuanha. It was found necessary to add a quantity of salicylic acid equal to one-thousandth part of the weight of the sugar in the syrup. Syrups thus prepared were kept simply covered with a sheet of paper at a mean temperature of about 17° C. At the end of two months they were intact, whilst the same syrups, placed in the same conditions, but without salicylic acid, were completely altered.—*Ph. Jour.*

**Acute Rheumatism.**

In the *Currier Med.* J. W. Futrell recommends the following to be given in acute rheumatism: One ounce each of Epsom salts, nitrate of potash, and powdered sulphur are to be added to a quart of boiling water. After being allowed to stand in a covered vessel for six hours, the mixture is to be strained and given in doses of one ounce, three or four times a day. As a local application to the inflamed joints, he employs, at the same time, a liniment composed of olive oil, ℥ v.; chloroform, ℥ ij.; hartshorn, ʒ vj.; tincture of aconite root, ʒ ij. M. Apply sufficiently often to relieve pain, and give bromide of potassium at night to secure rest.

**Chloral Cream.**

The following formula is given by the *Repertoire de Pharmacie:*

Finely powdered sugar, 100 parts.
Hydrate of chloral, 5 parts.
Water, 15 parts.

Dissolve the hydrate of chloral in the water, and add the sugar. The mixture may be flavored with oil of peppermint or artificial essence of pineapple. After a while a liquid layer separates on top, containing a larger proportion of chloral than the lower portions, which mainly consist of undissolved sugar. The mixture, therefore, should be well stirred up before being administered.

**Syphilitic Buboes.**

A Dr. Jacubowitz, who writes to *Der Praktische Arzt*, in the treatment of syphilitic buboes injects a solution of iodide of potassium, in the proportion of one part to thirty of water; and he gives two cases in which his results were extraordinarily successful. In one case he made a puncture into the most prominent part of the gland which was enlarged to the size of a goose's egg, pushing in the needle obliquely to a considerable distance. After injecting about the fourth of the syringeful a resistance was felt; he then withdrew the needle for a short distance, penetrated the septum on one side, and again injected a quarter part. By repeating this process, he threw in about fifteen grains of the iodide in an ounce of water. The tumor almost immediately became harder, smaller, and less painful. After four injections, performed in the course of two days, the tumor gradually dwindled away to the size of a hazle-nut, and ultimately vanished altogether. The recent case was very similar. Here, however two dark blue bodies, remained, which were so hard that injection of them appeared to be impossible. It was, however, accomplished with entire success; ten injections being required altogether. The small quantity of the iodide required is quite notable.—*The Practioner.*

**Chloral Plaster.**

Dr. Solari, of Marseilles, recommends this plaster as an excellent application in cases of neuralgia and certain nervous pains arising from exposure to cold. The plaster is easily prepared by powdering the chloral over a common pitch plaster—one to two scruples of chloral for every four square inches of plaster. Care is taken not to incorporate the chloral with the pitch. It is applied for twenty-four to forty-eight hours; when removed, the skin is found covered by a number of small vesicles; these are opened, and the part then covered with a cerate dressing. Generally speaking, it will be found that the pain has disappeared before the vesicles heal. Dr. Solari states that numerous cases of lumbago, intercostal and other forms of neuralgia, etc., have been rapidly cured by this simple method.—*Paris Medical.*

**Poisoning by Rhus Toxicodendron.**

Dr. T. D. Williams, of Chicago, writes to the *Druggists' Circular:* In the fall of 1873, while gathering herbs for pharmaceutical purposes, I accidentally brought my left forearm into contact with the end of a freshly cut shoot of poison oak. Not being, as I thought, susceptible to its toxical effects, I paid but little attention to the occurrence. A few hours afterwards, during which time an intense itching sensation was experienced, I noticed the usual tumefaction and rapidly appearing erysipelatous rash. Having had considerable experience in the use of hyposulphite of soda in various forms of disease, I concluded here was a test for its antidotal power, if it possessed any, and I at once applied a saturated solution to the affected part. The effect was marvelous, positively magical, as in three or four hours' time every symptom of the disorder had disappeared. Since that time I have used it with like result, not only in case of rhus poisoning, but also in erysipelatous inflammation. Both internally and locally it proves itself a reliable remedy. Excepting the one equivalent of sulphur together with the two equivalents water, hyposulphite of soda does not differ from the sulphite of the same base. Therefore, from the experience, as related above, I must believe, if Dr. Morrison's statement in relation to the effect of sulphite of soda is correct, that the additional amount of sulphur, in form of hyposulphurous acid, is the reason why hyposulphite of soda is preferable for the local treatment of disease and rhus poisoning.

**Asthma.**

Dr. Reeves states that smoke derived from the leaves of belladonna possesses much more power in cutting short an attack of asthma than that from stramonium. A long pipe is the best means of smoking them, the patient being instructed to draw the smoke deep into the chest. If when the attack is at its height, he has not the power of doing this, the leaves may be placed in a saucer containing lighted charcoal or wood-ashes, which should be placed on a chair in front of the patient—this chair as well as his own, being covered with a large sheet, so as to confine the fumes before the leaves are put on the hot charcoal. From two and a half to five grains of the leaves are sufficient when smoked, and from five to twenty grains when burned. If the smoke is drawn deeply into the chest, the relief is immediate, expectoration of phlegm taking place. It is in the spasmodic form of asthma, where there is little or no expectoration, that the greatest relief is obtained; for, when the tubes are loaded with mucus, the smoke cannot get access to their muscular tissue. The relief is most marked when the remedy is used soon after the paroxysm has commenced, before the spasm prevents access of air to the smaller tubes and air-cells. Tobacco-smokers do not receive the same amount of relief as others. Temporary headache of a throbbing character may be produced if the leayes are used too freely.—*Melbourne Med. Record,* May 24.

**Instantaneous Sinapisms.**

Dr. Rigabert describes a sinapism invented by M. Vincent, a chemist at Saintes, which, he says, for promptitude and certainty surpasses Rigollot's papers. These last are apt to deteriorate by keeping, and at all events require the aid of water to produce its effects. M. Vincent's procedure is as follows: Into a tube, open at the end, five centimetres in length, and having a calibre of half a centimetre, he pours a certain quantity of freshly prepared essence of mustard; the tube is corked and hermetically closed, and is wrapped up in a piece of paper of tolerable consistence of the size of one of Rigollot's sinapisms. When it has to be used some drops of the essence are poured on the paper, which is applied as an ordinary sinapism. The effect is instantaneous and certain; and by pouring on the paper the contents of two tubes at the same time, vesication can be produced. —*Bull. de Therap.*

**Embalming.**

Dr. G. Bufalini, (*Ann. Sc. ed. Industr., IX., Milan*) has found that carbolic acid, combined with camphor, is excellent for the preservation of anatomical specimens. He prepares the mixture by bringing crystals of carbolic acid in contact with pieces of gum camphor. The crystals unite and form a substance resembling oil. He dissolves the whole in a sufficient quantity of petroleum, previously colored with cinnabar, in the following proportions: Two hundred grammes of petroleum, seventy grammes of carbolic acid and camphor; or better, one hundred and thirty grammes of camphor and carbolic acid, and one thousand grammes of petroleum. The mixture may serve as well for injections as for the immersion of pieces that are to be preserved. The pieces harden, but become soft and flexible when they are plunged into warm water. This mixture is not dangerous, and does not injure or stain the instruments.—*Detroit Rev. of Med.*

**Sore Nipples.**

In an article published in the *Currier Medical*, Dr. Charrier recommends the use of picric acid in solution as a cure for sore nipples. The application has the effect of rapidly removing the pain, and checking the morbid secretions. The acid is used in the following manner: It is important to employ the chemically pure product, perfectly free from soda, and two solutions of different strength are prepared. The first, or *concentrated* solution, is made of three drachms of picric acid dissolved in two pints of water; and the second, or *weak* solution, only contains sixteen grains of acid to the same quantity of water.

To apply the lotions, the nipple is first well cleaned and washed with a fine sponge and lukewarm water; then a very fine camel's-hair pencil, dipped into the concentrated solution, is gently passed over the chapped and inflamed parts. This is done once a day, in the morning; and each time after the child has been suckled, the nipple is immersed during three or four minutes in a small glass filled with the weak solution.

After from twelve to twenty-four hours, the sharp pains experienced when the child is suckled begin to disappear, and all the red and inflamed parts assume again their natural color. The picric acid solution extinguished, as it were, the inflammation on the spot, prevents the lymphangitis from extending further, and therefore averts phlegmons and abscesses of the mammæ. Another advantage of the acid is that it tans the thin epidermis of the breast, rendering it thereby less liable to alterations.

**Diabetic Subjects before Surgical Operations.**

Dr. H. Fisher, of Breslau, in view of the unfavorable results which attend operations upon diabetic subjects, has lately resorted to the use of carbolic acid internally for the reduction of the amount of sugar, as suggested by Ebstein and Muller, and says that if this agent be administered internally in small and frequently repeated doses, it will, in a short time, cause considerable reduction in the amount of sugar in the urine, and permit the surgeon to perform his operation with the ordinary chances of success. The use of carbolic acid should be kept up during the after-treatment, and not be discontinued until the wound, formed in the operation, is quite closed. In cases of severe and extensive inflammation following an important operation in the subject of overlooked diabetes, the author has frequently succeeded in arresting the local mischief by reducing the amount of sugar in the urine through the administration of carbolic acid. He has also observed good results from an energetic administration of carbolic acid in cases of the so-called diabetic carbuncle.—*London Med. Record*, from the *Deutsche Med. Wochenschrift.*

**Polyuria.**

M. Hayem mentioned at a meeting of the Paris Biological Society, the case of a man aged forty-eight, who had enjoyed good health up to 1867, when he suffered from a nervous attack, loss of consciousness, and paralysis of the right arm. In 1874 the sight became impaired. The patient remained in good health up to February, 1875, when he became afflicted with polyuria, polyphagia, and polydipsia, soon connected with nocturnal incontinence of urine. He was ordered issues to the lumbar region, and iodide of starch. When the patient was first seen by M. Hayem the face was puffed and the lower limbs œdematous. The urine did not contain any albumen, but a certain amount of sugar. He voided in the twenty-four hours four quarts and a half of urine, half an ounce of urea per quart—viz., about two ounces altogether. After a treatment of twenty-eight days, the patient taking about one grain of opium per diem, the quantity of urine diminished to about three pints per day, and the urea to less than half an ounce per quart—viz., seven drachms in the twenty-four hours. The general health was much improved.

---

**Intestinal Obstruction.**

Dr. Brun, in a communication to the *Journal de Medicine* of last month, describes a case of intestinal obstruction, due to the ingestion of large quantities of preserved fruit, which was successfully relieved by means of J. Wood's method of insufflation of air. The difficulty had lasted for four days, and had resisted all the ordinary methods of treatment. Moreover, the procedure recommended by Taliaferra, of injecting into the bowels bicarbonate of soda and citric acid, separately, for the purpose of liberating gas in the intestines and distending the canal, had been tried ineffectually. The patient was evidently sinking; the pulse was very feeble, and there was stercoraceous vomiting. An œsophageal sound was introduced into the rectum, and to the external extremity the nozzle of an ordinary bellows was attached. Air was then injected till the abdomen had increased markedly in volume. The first attempt, however, was unsuccessful. It was repeated, and the insufflation continued until considerable abdominal tension was produced, and respiration was rendered difficult. In the course of an hour dejections began and the patient recovered.

---

**Burns.**

Dr. Bedford Brown, in an article on burns, published in the *Philadelphia Medical Times*, recommends the following treatment to allay pain and promote the process of healing:

Take iodoform, 2 dr.
Spermaceti ointment, 1 oz.
Ext. of conium, 1½ dr.
Carbolic acid, 10 drops.

This, spread on fine linen, is applied twice daily to the inflamed surface, and then enveloped in oiled silk, no other dressing being required. In cases where there is great dryness of surface from destruction of vitality and want of exhalation, the wound, before applying the ointment, should be coated with the common linimentum calcis, which affords a soft and moist dressing, and in no wise interferes with the action of the iodofrom. The iodofrom acts as a certain and most effective sedative on the painful and exposed surface, and at the same time as an antiseptic. It reduces inflammation and suppuration, when in excess, in a remarkable manner, promptly converting a most painful and irritable wound into one that is comparatively painless. It is also an excellent promoter of healthy action and the healing process, and has besides the great advantage of rendering the use of anodynes unnecessary.

---

**Whooping Cough.**

Dr. Sawarowsky writes to the *St. Petersburger Med. Wochenshrift*, Sept. 30th, recommending the inhalation of a mixture containing f ℥ j. of chloroform and three drops of nitrite of amyl. Besides this he administers for three days, at intervals of two hours, the following powder: Argent. cyanidi, gr. 1-12; Argillæ puræ, gr. iij. Hot articles of food are to be prohibited. Under this plan of treatment the most obstinate cases have yielded within a very few days.—*The Clinic.*

ANOTHER.

According to the *Cincinnati Lancet*, the following formula will relieve whooping-cough: "Narceine, 25 centigrammes; simple syrup, 500 grammes, and a few drops of acetic acid. Each tablespoonful will contain 1 centigramme, and half a tablespoonful can be given for a dose. In the course of twenty-four hours two or even three centigrammes can be given.

ANOTHER.

In a paper read by Dr. P. Brynberg Porter before the New York Medical Journal Association, and published in the *New York Medical Journal*, of October, the author detailed the results of using chloral, quinia, castanea, and inhalations of ether in the treatment of pertussis. His conclusions are, in the main, like those arrived at by him in a former paper, (1873) and may be summarized as follows: It is his opinion that in the average number of cases where either of the three remedies first mentioned seems to be of advantage and is given early, the paroxysmal stage is about three weeks in duration. He has found castanea to fail in a larger proportoin of cases than either chloral or quinine. He thinks that with one or the other, or perhaps two of these remedies together, we ought not to find pertusses to be such a "formidable adversary," as it has heretofore been generally considered.

---

**Rickets.**

We give the following nearly *verbatim* from the *Centralblatt fur Chirurgie;* it is taken from the *Gazette Hebd.* The idea suggests a well-known circumstance that is said to have occurred some time ago at the Zoological Gardens. A lioness was in the habit of throwing litter after litter of young cubs with cleft palates, until it was found that the substitution of a more bony diet, in the shape of whole rabbits, during gestation, for the more refined food which the Society provides, had the effect of remedying this serious defect. In the same way it appears that the nourishment provided by nature for the lords of creation is not the best suited to some of his inferiors, and that even for him under some conditions, a coarser diet is beneficial. The notion of feeding children on dog's milk is not a pleasant one: the objection is, of course, sentimental; but surely an artificial fluid might be made to answer the purpose equally well? It is the custom amongst the women of Monttrun, in Dauphine, to continue suckling for two and a half or three years, with the idea of preventing another pregnancy; and if the infant dies the mother either adopts another, or takes a puppy into her family to carry on the process. All these puppies suffer from rickets, which resembles exactly the rickets of children, except that the deformity is never afterwards remedied. These observations, and the fact that the dogs always recover under the influence of their own mothers' milk, induced M. Bernard to submit a rickety female child of twenty-six months to the dog's-milk cure. A powerful bitch was provided to act as wet-nurse for the child, and after from two to three months of this new method of imbibing nourishment, the swelling of the epiphyses and the bending of the bones had notably diminished, the muscles were stronger, and at the end of the time the child could stand and take a few steps. The health of the patient was at the end of one hundred days extremely good, a slight curve of the femur and sternum being the only remains of the deformity, and the cure was permanent. He has adopted the treatment successfully in six other cases, and he expresses the belief that it will give encouraging results.

---

**Bromide of Arsenic in the Treatment of Epilepsy.**

Dr. Th. Clemens, of Frankfort-on-the-Main has employed bromide of arsenic for twenty years in the treatment of diseases of the nervous system, and especially of epilepsy, and claims that he has obtained astonishing results with it. He uses the liquor of bromide of arsenic, and gives one or two drops in a glass of water once, or, if necessary, twice daily. These minute doses may be given for months, and even years, without producing the usual unpleasant effects of a long-continued arsenical course. All his cases of epilepsy have been markedly relieved and improved by this remedy, but only in two cases has it produced a complete cure. In many cases of incurable epilepsy, complicated with idiocy and deformities of the skull, the fits were reduced in number from twenty in the twenty-four hours to four or even two—a result that has been obtained by no other treatment. In connection with the bromide of arsenic, an almost exclusively meat diet is advised. The patients should be as much as possible in the open air in daytime, and their windows should be kept open at night. Unlike bromide of potassium, this remedy does not require to be given in increasing doses, and instead of interfering with digestion improves the nutrition and strength. Dr. Clemens has employed the following formula since 1859, and thinks that it ought to replace Fowler's solution, which is irrational in its composition and uncertain in its action. This solution becomes stronger with time, the chemical union of the bromide with the arseniate of potash becoming more and more perfect.

LIQUOR ARSENICI BROMIDI.

Arsenious acid, powdered, 1 drachm.
Carbonate of potassa 1 drachm.
Bromine 2 drachms.
Water, enough to complete 20 ounces.

Boil the acid and carbonate together until the dissolution is effected, add enough water to complete the quantity, and the bromine when the mixture has become cold.

---

**Cystitis.**

Dr. Wm. Semple says most of the cases of acute cystitis that have come under his observrtion have occurred in young girls with whom the menstrual function had not become regularly established, and the attacks have commenced soon after a menstrual period; and in unmarried women when the function, before its cessation, becomes irregular. He has not found occasion to resort to the introduction of instruments into the bladder for purposes of examination or treatment since he has adopted the method here recommended. This method consists in the administration by enema into the rectum of from forty drops to a drachm of a solution of sulphate of atropia (one grain to eight ounces of water), to which is added sufficient carbolic acid to prevent the formation of organic matter and the deposit of atropia. The dose is added to half an ounce of water for administration, and given twice in twenty-four hours. It uniformly and immediately arrests the frequent strangurious and painful micturition, gradually checks the mucous and sanguineous discharges, and relieves the supra-pubic pain with the cystic inflammation. When the urine is alkaline, Mettauer's nitro-muriatic acid is given to correct it; and when it is so acid as to irritate, the acidity is corrected by antacid remedies, of which the bicarbonate of potash, with subnitrate of bismuth, is generally preferred, because of the tonic effect of the bismuth and its very soothing effect on the mucous surfaces of the urinary organs. When constipation exists, which is frequent, it is relieved as occasion requires, generally by the German pulvis glycyrrhizæ compositus until the bowels begin to act regularly from the effect of the atropia, which generally soon results. Several cases are reported to illustrate the success of this method of treatment.—*Virginia Medical Monthly.*

**Gonorrhœa.**

Radha Nauth Roy, Assist. Surgeon, extols (*Indian Medical Gazette*, May, 1857) the efficacy of injections of quinia in gonorrhœa. He states: "I was once tempted to try it in a case of acute gonorrhœa, where scalding was unbearable and discharge profuse, and to my utter surprise after the third day, I found the man quite relieved. He described to me the soothing effect of the injection as something cold, like ice. The discharge was so much diminished that his cloths were scarcely stained after the third day. There was no more incessant desire to void the bladder, and he was to all appearance comfortable. My success in this case made me bold enough to use it in other cases, and I have invariably found the disease yield both in its acute and chronic stage under its influence. It acts as a tonic and astringent to the mucous membrane of the urethra. I have also used it in some cases of cystitis with much benefit. I generally use it dissolved in sulphuric acid, diluted, mixed with rose water. Two grains of quinine sulph. dissolved in acid sulph. dil. m. viij. or m. x. and mixed with an ounce of rose water; to be used twice for injection. At the same time I gave copaiba mixture to my patients. In almost all the cases I have found it act like a charm. The disease is generally cured within a week, but chronic cases take a longer time. In a few acute cases it took more than a fortnight, but the delay in them was attributable to their irregular habits durthe treatment."—*The Clinic.*

**Puerperal Convulsions.**

Dr. Chouppe, having had the opportunity of observing carefully a considerable number of cases of puerperal convulsions, has come to the conclusion that, of all the means we possess, hydrate of chloral is the most reliable for treating this disease. In twelve cases in which it was alone employed the termination was successful, although in some of these the state of things looked desperate when it was commenced. He thinks, indeed, that it should be resorted to even before the disease becomes confirmed, whenever the woman, exhibiting albuminuria and œdema, complains of headache, singing in the ears, hallucinations of vision, restlessness, cramps or vague pains in the limbs, etc. When there is trismus present it should be given in enemata, which have also the great advantage of being able to be given during the paroxysm. The doses will vary according to the tolerance of the patients and the severity of the paroxysms, but it is necessary to commence with a pretty strong one (especially if the paroxysms are violent and close upon each other), in order to make a powerful and quick impression. After a calm has been obtained, and if the attacks do not recur, some smaller doses may be given during the next twenty-four hours or so; but if the attacks recur, larg er doses must again be resorted to until the parox ysms have completely ceased. In an enema we may always begin with thirty grains, repeating this at the end of ten minutes; and by the mouth at least forty-five grains should be given at once, fifteen grains being repeated every quarter of an hour. In a violent attack the dose required will vary from 120 to 180 grains; and it may even be requisite to resort to hypodermic or intravenous injection. In all cases it is of importance to get at least sixty grains rapidly taken, and to prolong the use of the chloral for a tolerably long time after the cessation of the convulsions.—*Gazette Med.*

**Glandular Swellings.**

A new use for alcohol has been discovered by a German physician of Weinheim, Dr. Schwalbe, who, if we may credit the accounts which reach us, has reported one hundred cases of various forms of tumors and glandular swellings which he has successfully treated by the subcutaneous injection of the tincture of iodine. Subsequently having injected the simple alcohol with equally favorable results, he concluded that the latter is really the effective agent. The subcutaneous injection of this fluid produces chronic inflammation of the connective tissue, by which it is caused to contract, and the pressure thus produced obliterates the lymphatics. In some cases he has used ether in connection with alcohol in order to dissolve fats, and in this way he reports that he has cured several cases of fatty tumors. But the most important discovery of all is that alcohol thus administered prevents the extension of the cancer to neighboring tissues and lymphatic glands. By injecting the alcohol the tumor may be isolated, as it were. The contractive connective tissue then approaching the growth itself, presses upon it, stops the supply of blood and causes it to disappear by atrophy. In connection with Dr. Hasse, Dr. Schwalbe claims that he has cured three cases of cancer of the breast in this way. We suppose that temporary cures are meant, as cancer is a disease of the blood, and is, therefore, constitutional, although locally manifested; and it is well known that the successful removal of cancer of the breast is very apt to be followed by a re-appearance of the disease elsewhere.—*American Artisan.*

---

**Erysipelas.**

In the London *Medical Times and Gazette*, Dr. Clarence Foster recommends the following treatment:

I wish to direct the attention of my medical brethren to the immense utility of the tincture of iron, locally applied, in arresting erysipelas and many other external diseases when unattended by breach of surface. In simple cutaneous erysipelas, and also in the milder phlegmonous variety, it possesses the decidedly specific effect of subduing, almost at once, the morbid action. I have applied it in numerous instances, and always with the most satisfactory results. So far as my experience goes, it is in these cases incomparably the best external remedy ever used. It seldom happens that more than one painting of the same spot is required; and, having applied it, no other external agent whatever is needed. In scrofulous swellings of the neck, its discutient properties are far superior to iodine; and where a puerperal breast or inguinal gland in the male has threatened to end in suppuration, the early use of the tincture, every other day or so, with a camel's hair brush, has been sufficient to effect resolution, while in similar cases we find frequently that leeches, poultices and evaporating lotions, fail to prevent the formation of matter. Again, the remedy may be applied most advantageously in acute rheumatism, where any particular joint is swollen and painful, and also to the inflamed surface surrounding an unhealthy ulcer, or along the course of the absorbents when irritated by a recent, ill-conditioned wound. The well-known remedy, ink, as a domestic application in ringworm, has long enjoyed a not altogether undeserved popularity, its curative effect being undoubtedly due to its ferruginous ingredient. Although the external use of the tincture of iron—first introduced by my father, I believe, some five-and-twenty years ago—is now pretty common in the West Riding, yet its great therapeutic advantages, I have reason to think, are far from being sufficiently appreciated by the profession generally, and I am fully convinced that any surgeon giving the preparation a trial will be amply satisfied with the result.

---

**Selected Hospital Formulæ.**

Standard formulæ used at Bellevue or Charity Hospital, New York City:

STOKES' EXPECTORANT MIXTURE.

℞

Ammon. Carb., gr. 32.

Ext. Fl. Senegæ, } āā fl. ʒ 1.

Ext. Fl. Scillæ, }

Tr. Opii Camph., fl ʒ 6.

Aquæ, fl ℥ ½.

Syrup. Tolut., q. s. ad fl. ℥ 4.

Teaspoonful. M.

MIST. HYDROCYANICA. CH. H.

℞

Potass. Cyanid., gr. 2.

Vin. Antimon., ʒ 2.

Syr. Tolut., } āā fl. ℥ ½.

Mucil. Acac., }

Aquæ, q. s. ad fl. ℥ 2.

Teaspoonful. M.

MIST. LAFAYETTE

℞

Bals. Copaiv., } āā ℥ ½.

Spts. Æther. Nit., }

Liq. Potass., ʒ ½.

Spts. Lavand. Co., ℥ ½.

Mucil. Acaciæ, q. s. ad ℥ 4.

Tablespoonful. M.

MIST. ASTHMATICA.

℞

Spts. Æth. Co. } āā ℥ 1.

Sol. Morph. U. S., }

Tablespoonful. M.

MIST. BUMSTEAD.

℞

Bals. Copav., fl. ʒ ½.

Tr. Ferri Mur., } āā ʒ 2.

Tr. Cantharid, }

Syr. Acaciæ, q. s. ad fl ℥ 4.

Tablespoonful (in Gonorrhœa). M.

MIST. DIARRHŒA.

℞

Tinct. Opii, }

" Camph., }

" Capsici., } āā p. e.

" Menth. Pip., }

" Rhei Arom., }

30 to 40 drops in water. M.

MIST. BINIODIDI.

℞

Hydrarg. Bichlor, gr. 1.

Potass Iod., ʒ 2.

Tr. Gent. Co., fl ℥ 2.

[Tr. Cinch. is less proper, because some of the alkaloids are precipitated with iodohydrargyrate of potassium.]

Teaspoonful. M.

**Sea-Sickness.**

Dr. Crochley Clapham, believing the proximate cause of sea-sickness to be an undue congestion of the vessels of the spinal cord, has tried to remedy this condition by the use of nitrite of amyl in one hundred and twenty-four cases; one hundred and twenty-one of which proved eminently satisfactory, there being no return of the malady after the administration of the nitrite. The remaining three cases were only unsuccessful in so far as they required a further dose or two of the remedy.

The mode he adopts of exhibiting the drug is by inhalation, three drops of the nitrite being poured on a handkerchief and held *close* to the patient's nose. The inhalation must be conducted rapidly, so as to give the full influence of the drug without a *too free* admixture of air.

The action of the remedy in freeing the circulation and relieving the hyperæmia of the spinal cord will be quickly evidenced by a throbbing sensation in the temples (occasionally rather disagreeable) and by a more or less general flushing and increased warmth of the surface of the body. This warm and comfortable glow, which takes the place of the chilly sweat so disagreeable in this disease, is usually followed in course of half an hour by a pleasant slumber, from which the patient wakes to eat a hearty meal. Should the sickness recur, which it may do after the lapse of twenty-four hours, the inhalation must be repeated. The patient should be in bed when under treatment, so as not to interfere with the subsequent sleep; and it is usually better to allow one fit of vomiting to take place before applying the remedy, not only to insure the bona fide character of the seizure, but also because it is advantageous unless the patient be in a very weak state of health. He met with only one case in which the medicine was refused on account of its disagreeable effects, and in this instance, which occurred in the tropics, the patient complained that "it made him feel so hot he would rather be sea-sick."—*Phila. Med. Times*, from the *Lancet.*

---

**Primary Diseases of the Heart.**

Before the Harveian Society of London, lately, Dr. J. M. Fothergill read some notes of cases of these diseases. The line of treatment pursued in the cases was rest, at first, and the steady administration of digitalis and iron. The administration of digitalis might be continued for years, uninterruptedly, without the production of those toxic symptoms which were supposed by older writers to indicate some cumulative action in this drug. As well as acting directly upon the heart, in advanced cases with dropsical effusion, Dr. Fothergill spoke strongly in favor of the use of cathartics to relieve the venous congestion. He gave a case where two scruples of compound jalap powder were given every alternate morning, till eight doses had been taken, with excellent effects. The depressing effect of free purgation is more than compensated by the relief afforded in these cases. Digitalis and iron were also given, and the catharsis was only supplementary to the direct treatment of the heart itself. To illustrate what might be attained by such direct treatment of the heart, Dr. Fothergill abduced a case of mitral regurgitation in a young man, in whom a murmur could no longer be heard, and the subjective symptoms of disease of the heart had also vanished. Here the vela of the mitral valve were injured, and when the left ventricle was dilated, the injured valves were no longer equal to closing the ostium on the ventricular systole. The reduction of the ventricle to its normal size has resulted in the valves being once more competent; and as long as the ventricle can be maintained in a normal and undilated condition, the equivalent of a cure is attained. In mitral disease the use of digitalis is almost universally admitted, but there is less agreement as to its use in aortic disease. In Dr. Fothergill's opinion, its utility in aortic stenosis was obvious. In aortic regurgitation, in the early stages, it was contra-indicated, and an agent of precisely opposite qualities, one that would lessen the force of the ventricular contraction, and at the same time increase the number of beats—should be adopted, if we possessed such an agent. In the latter stages, however, when the muscular walls were failing, and death threatened from cardiac syncope, then digitalis was useful as a palliative agent. Valvular disease of the right side of the heart, and especially tricuspid disease, was little amenable to treatment, because no muscular hypertrophy could be brought to bear on it. Dr. Fothergill summed up the treatment of primary diseases of the heart as follows: 1. It is of the utmost moment in these cases to reduce the demand upon the heart to a minimum. 2. Much relief may be afforded, where dropsy is present, by unloading the congested venous system; and for this end cathartics are very serviceable. 3. The heart must be acted upon directly, by means of agents which increase the vigor of the ventricular contractions, of which digitalis is the chief. 4. To improve the general condition by the use of chalybeates and suitable food is also very desirable. Digitalis and iron may be continued for years, not only without any evil consequences, but with much advantage in many cases.

**Relief of Pain.**

Dr. S. Henry Dessau, of this city, reports in the *New York Med. Jour.*, of June, seven cases in which he followed the suggestion of Dr. Lelut, (*L' Union Medicale*,) in the use of cold water, introduced hypodermically, for the relief of painof a rheumatic character. In all the cases the operation worked well.

---

**Chronic Gastro-Intestinal Catarrh.**

S. C. Dumm, M. D., used chlorate of potassium in a case of chronic gastro-intestinal catarrh, attended with inflammation of the bile duct, with excellent results. Ten grains were given every three hours, dissolved in water. After the use of the remedy for one day, pain in the stomach ceased, the appetite improved, vomiting stopped, and the patient recovered her health quickly.

---

**Erysipelas.**

John Firnat, M. D., reports cases and says: "After having tried tincture of iodine, camphorated ether, nitrate of silver, carbolic acid with oil of turpentine, acetate of lead ointment, oxide of zinc ointment, and styptic collodian, in erysipelas, in connection with internal treatment, I have found nitrate of lead with glycerine to give far more satisfaction. . . . I have also used the nitrate of lead, 10 to 15 grains to ℥ j of glycerine, in eczema of the head, washing previously with tar soap, and then applying it with entire success, in connection with small doses of Fowler's solution and iodide of potassium.—*Medical Times.*

---

**Ergot of Rye.**

M. Hayem reports, in the *Revue de Therapeutique*, a trial of ergot of rye in cases of enteric fever, with the object of lowering the temperature. The results he has obtained, have been very satisfactory, and its employment in this disease seems to him preferable to that of sulphate of quinine or of digitalis. Under the influence of ergot there is much more rapid defervescence; and at the period of the acme, instead of there being a rise in the temperature chart, a plateau is obtained. In some cases, in which the ergot was only given during the day, the evening temperature was not so high as the morning. The dose varied from thirty to fifty grains in the twenty-four hours.

---

**Solution of Glycerine and Water**

M. Muller, a pharmacist at Breslau, gives the following:

Salicylic acid, 1 gramme.
Glycerine, 20 grammes.
Distilled water, 80 grammes.

First treat the acid with the glycerine and then add the water.

In Switzerland salicylic acid has been used in typhoid and paludal fevers, etc. It has been noticed that it has a very remarkable cumulative action; for after having obtained the desired remission by a first dose of four to eight grammes, it has been found that a dose of one-half or one-fourth that quantity on the following days is sufficient to keep the temperature within good limits. Dr. De Cerenville recommends that these doses should be administered in water, flavored with liquorice juice.

---

**Necrosis.**

Dr. Ephraim Cutter reports in the *Boston Medical and Surgical Journal*, a case, with cure, of necrosis of the alveolar process, treated by injections of aromatic sulphuric acid, one drachm to the ounce of water. By means of a half-ounce syringe; supplied with an ivory tip, one inch and a half long, and one-eighth inch in diameter, the acid solution was injected at first twice a day, and afterwards once a day. About two drachms were used at each injection. The syringe tip was deeply buried in the soft tissues through one of the openings. Pus would freely exude from the other openings, even from one in the top of the mouth, after each injection. Tonics, with a diet of animal food and unbolted wheat, were administered. A marked improvement in the soft tissues occurred. In about forty days teeth, which had been loose, became solidly fixed in their sides. A few spiculæ of bone were removed from the front of the alveolar process during the period of treatment.

Dr. Atkinson, of New York, has reported some remarkable instances of cure of necrosis by this agent used in its full strength, it is said. It hastens the disintegrating and separating process, and at the same time destroys the germs of parasitic micrographic growths in the dead and dying bone. According to Dr. Atkinson, it does not act unhealthily on sound tissues, whose vital connections are unimpared.

---

**Diabetes Insipidus.**

Dr. Laycock, of Edinburgh (*Lancet*,) points out that both forms of diabetes, as well as certain kinds of Bright's diseases, are really *neuroses*, having their seat in that part of the encephalon which regulates the amount of water in the blood, and has therefore both anatomical and functional relations with the sudoriparous glands and the kidneys, and with the appetite for water and the sense for thirst. He now relates two cases of diabetes insipidus or polydipsia in which jaborandi was given with good effect. In the first case the quantity of urine passed was very great, amounting to 400 and 500 ounces per diem, and the patient was compelled sometimes to micturate every half hour. The urine was pale, almost colorless, faintly acid, of sp. gr. 1005, with no sugar or albumen, and a very small amount of the ordinary solids. He was under observation and treatment from December 24, 1874, to February 26, 1875, when there was still great thirst and the skin was dry, and the daily amount of urine voided was 300 ounces. Jaborandi was now ordered in the form of an infusion (one drachm of the leaves and twigs to six ounces of water,) and a dessertspoonful was taken every four hours, this dose being increased and given at shorter intervals on succeeding days. On March 5, or about a week after the jaborandi was given, the skin of the back, abdomen, and inner aspect of the thighs was found to be perspiring pretty freely, and on the 6th the skin of the arms and left palm perspired. On the 15th the quantity of urine had declined steadily from 300 ounces to 236; and on the 21st, the jaborandi treatment having been still continued, the quantity fell to 180 ounces. The urine continued to diminish in amount till the middle of May, when it amounted to 120 ounces a day, and the patient was then discharged at his own request. In the second case the daily quantity of urine passed was 128 ounces, the sp. gr. being 1008, of acid reaction, and containing some albumen, but no sugar. There was great thirst, and in order to quench it the patient was obliged to drink a large quantity of water at a time. This patient was placed at once on the jaborandi treatment, one tablespoonful of the decoction being given thrice daily. The quantity of urine passed and of fluid drunk in twenty-four hours was carefully noted, and the results were that the amount of urine fell in about two months to 98 ounces, and that of fluid drunk fell to 100 ounces, the amount at first being 186 ounces.

---

**Diarrhœa.**

Dr. E. T. Comegys, of the U. S. Army, stationed at Fort Duncan, Texas, sends the following communication to the *Medical Record*. It is interesting at the present time, when ergot is attracting so much professional attention, on account of the numerous successful and various therapeutical uses to which it has recently been applied.—He says:

Having had marked success with fluid ext. ergot in the treatment of diarrhœas, I have thought it well to publish the results of my cases, in the hope that others may give it a more extended trial than my limited facilities have enabled me to do.

I have based my use of this drug on the theory that it causes a contraction of the involuntary muscular fibres, and in so doing relieves the atony of the vessels of the intestinal mucous membrane and the consequent hyperæmia of the capillaries (which is always associated with diarrhœas,) and thus prevents the transudation of the watery portions of the blood. In other words, it gives *tone* to the vascular walls. Be the theory worth what it may, the success attending its use has been marked. I first tried it (in my clinic at the Dispensary of the Cincinnati Hospital) in the case of a patient with chronic diarrhœa of two years standing. No line of treatment had been able to reduce the number of his stools to less than eight per day. All other treatment was stopped, and he was given forty drops of the ergot four times a day. He came back four days afterward, saying that he was only having two stools per day, and when he next reported, (ten days afterwards,) he said that he had been entirely well for the past eight days. I heard from him three months afterwards, and he had had no return of his diarrhœa. He used the ergot six days.

I used it frequently after that in ordinary simple diarrhœas (giving no other medicine,) and found that the patient seldom required beyond three doses (twenty drops each,) and never more than six.

While stationed at Fort Wadsworth, N. Y. Harbor, last summer, I used it in twelve cases (the number of stools varying from eight to twenty in the twenty-four hours,) and in but one case was I obliged to give more than three doses to each case. In that one case it failed to modify the diarrhœa, but that may be accounted for by the fact that the ergot had beeen kept on hand for a long time.

Since I have been stationed at this post, I have had but three chances to try it. In one case it failed (case of chronic diarrhœa of four years' standing); in the second one my patient refused to take more than one dose on account of its unpleasant taste; in the third case it succeeded.

I offer this with the hope that others may give it a more extended trial than I have been able to do, and that they may publish their results.

